# Synthesis of Cationic Quaternized Nanolevan Derivative for Small Molecule and Nucleic Acid Delivery

**DOI:** 10.3390/gels9030188

**Published:** 2023-02-28

**Authors:** Chonnipha Charoenwongphaibun, Chanchao Lorthongpanich, Prapasri Septham, Karan Wangpaiboon, Pawinee Panpetch, Rath Pichyangkura, Thanapon Charoenwongpaiboon, Kamontip Kuttiyawong

**Affiliations:** 1 Division of Chemistry, Department of Physical and Material Sciences, Faculty of Liberal Arts and Science, Kasetsart University, Kamphaeng Saen Campus, Kamphaeng Sean, Nakhon Pathom 73140, Thailand; 2 Siriraj Center of Excellence for Stem Cell Research, Department of Medicine, Faculty of Medicine Siriraj Hospital, Mahidol University, Bangkok 10700, Thailand; 3 Department of Biochemistry, Faculty of Science, Chulalongkorn University, Bangkok 10330, Thailand; 4 Center of Excellence in Structural and Computational Biology, Department of Biochemistry, Faculty of Science, Chulalongkorn University, Patumwan, Bangkok 10330, Thailand; 5 Department of Chemistry, Faculty of Science, Silpakorn University, Nakhon Pathom 73000, Thailand

**Keywords:** levan, fructan, exopolysaccharide, drug delivery

## Abstract

Levan is a biopolymer composed of fructose chains covalently linked by β−2,6 glycosidic linkages. This polymer self−assembles into a nanoparticle of uniform size, making it useful for a wide range of applications. Also, levan exhibits various biological activities such as antioxidants, anti-inflammatory, and anti-tumor, that make this polymer very attractive for biomedical application. In this study, levan synthesized from *Erwinia tasmaniensis* was chemically modified by glycidyl trimethylammonium chloride (GTMAC) to produce cationized nanolevan (QA-levan). The structure of the obtained GTMAC−modified levan was determined by FT-IR, ^1^H-NMR and elemental (CHN) analyzer. The size of the nanoparticle was calculated using the dynamic light scattering method (DLS). The formation of DNA/QA-levan polyplex was then investigated by gel electrophoresis. The modified levan was able to increase the solubility of quercetin and curcumin by 11-folds and 205-folds, respectively, compared to free compounds. Cytotoxicity of levan and QA−levan was also investigated in HEK293 cells. This finding suggests that GTMAC−modified levan should have a potential application for drug and nucleic acid delivery.

## 1. Introduction

Polysaccharides are a type of hydrophilic polymer that have an inherent biocompatibility and bioactivity. They are frequently used in water-based polymer systems as well as nanotechnology for a variety of applications including drug transport, tissue engineering, and drug carrier [1]. There are many benefits to using polysaccharide-based nanoparticles (NPs), including high stability, low toxicity, and rapid drug release [2,3]. The free carboxyl and hydroxyl groups found along the polysaccharide backbone are the primary targets for chemical functionalization. Using these reactive groups provides the route for the generation of polysaccharide derivatives with desirable characteristics, expanding the scope of polysaccharide application.

Exopolysaccharides produced from microorganisms are a structurally varied class of compounds. A number of them have found applications in a wide range of sectors, including food, feed, chemical, cosmetics, packaging, textile, and medicine. In the nature, they have an important function in protecting cells, allowing bacteria to adhere to solid surfaces, and facilitating communication between cells [4]. Microbial EPSs are economically competitive to plant and algal polysaccharides because they enable faster and high yielding production processes under controlled conditions. Microbial polysaccharides such as xanthan, dextran, and pullulan are examples of polysaccharides that have a massive market because of their special characteristics.

Fructan is a biopolymer consisting of a chain of fructose that covalently linked by β−2,1 or β−2,6 glycosidic linkage [5]. This biopolymer is resistant to the enzymes in human digestive system, but it is degraded by colonic bacteria, allowed the fructan to be used as prebiotics. Fructan was either found in plant or synthesized by some bacteria such as *Bacillus* spp. [6], *Leuconostoc* spp. [7], *Zymomonas mobilis* [8], and *Erwinia* spp. [9]. In comparison to plant fructan, microbial fructan could self-assemble into nanoparticles, suggesting a broad spectrum of applications [9,10]. For example, β−2,1 fructan derived from *Lactobacillus reuteri* 121 inulosucrase can improve the solubility, antioxidant activity, and stability of quercetin and fisetin [10]. Nakapong et al. displayed the application of β−2,6 fructan in encapsulation of vitamin E, O−acetyl−α−tocopherol using direct enzymatic synthesis and self-assembling methods [11]. Mutlu et al. developed levan−based NPs for the purpose of transporting the cancer drug paclitaxel (Taxol) [12]. Cinan et al. used electrohydrodynamic atomization technique to prepare the resveratrol-loaded levan nanoparticles with high biocompatibility [13]. Levan was used for targeted breast cancer imaging encapsulation of indocyanine green (ICG) within levan by self-assembled process [14]. In addition, several research demonstrated that levan type fructan also exhibited a variety of biological activities anti-tumour, anti-inflammation, wound-healing, regulation of cell proliferation and biocompatibility [15].

Besides a native form of fructans, chemical modification has allowed for a broader variety of fructan use. For example, sodium periodate (NaIO_4_) oxidizes levan to aldehyde levan, which can be utilized as a carrier for immobilization of proteins [16]. Phosphate-modified levan was assembled with chitosan to form the films [17]. Sulfated levan from *Halomonas smyrnensis* displayed anticoagulation activity via like heparin in a dose-dependent manner [18].

Polymeric drug delivery systems and gene delivery technologies have seen considerable developments in the past two decades [19]. Drug delivery technologies could provide several vital treatments such as control release, drug protection, solubility enhancement, target selection etc. However, limitation of most polymeric drug delivery system is low drug loading capacity. Therefore, researching new drug delivery systems and their impacts is still crucial. The use of polymers that have their own biological activities could make it possible for drug delivery systems to be more effective. In addition, several naturally occurring polymers have the ability to target certain organs or cells in a selective manner. Previously, levansucrase from *E. tasmaniensis* has been well characterized and is utilized in levan production [9,20]. The obtained levan has unique properties compared to other microbial fructans, for example; it could form gel or self-assemble into size-uniform nanoparticles (~157 nm), suggesting its versatility. Moreover, *E. tasmaniensis* levan significantly displays anticancer activity and immune stimulation activity [9,21]. Therefore, the use of levan as a drug carrier could enhance effectiveness of drug delivery system and might be a promising approach of treating patients in the future. Nevertheless, to the best of our knowledge, chemical modifications of *E. tasmaniensis* levan has not been reported yet. Chemical modification of polysaccharides can be required for various reasons, including to alter their physical properties, make them more stable and modulate their biological activities. Herein, levan synthesized by *Erwinia tasmaniensis* levansucrase was derivatized by glycidyl trimethylammonium chloride (GTMAC). Because GTMAC has a quaternary ammonium group and a hydrophobic tail, the modified levan would bind to negatively charged molecules and water-insoluble compounds. The structure of the chemically modified levan was analyzed by elemental analyzer, FT-IR and ^1^H-NMR. After that, ability of drug−binding and DNA polyplex formation of the cationized levan were investigated by spectrophotometer and agarose gel electrophoresis. The obtained results should be useful to expand the use of nanolevan in medical applications.

## 2. Results and Discussion 

### 2.1. Chemical Modification of Levan and Characterization

Levan is a type of microbial polysaccharide that is composed primarily of fructose and is joined together in the main chain by a β−2,6 glycosidic bond, and also contains some β−2,1 branch. It has been known for a long time that levan can be utilized in the delivery of drugs as well as in other medicinal applications. Previously, native levan was used to increase solubility of some small molecules, for example o−acetyl−α−tocopherol [11] and resveratrol [13]. Additionally, levan has undergone a process of chemical modification in order to achieve the desired improvements in its characteristics. Recently, levan hydrogel was prepared by cross linking with 1,4-Butanediol diglycidyl ether (BDDE) for controlled release of resveratrol [22] and Amphotericin B [23]. However, to the best of our knowledge, there have only been a few reports written about the preparation of cationized levan for the delivery of drugs and nucleic acids.

Glycidyltrimethylammonium chloride (GTMAC) was used to cationize various polysaccharides such as pullulan [24], arabinoxylan [25], chitosan [26], and starch [27] to develop nanocarrier for nucleic acid delivery application. In this study, GTMAC was grafted onto levan to produce a quaternized levan (QA-levan). Due to the fact that levan produced from *Erwinia tasmaniensis* can be formed into a stable nanoparticle with uniform diameter around 160 nm [9], it should have a potential application in drug delivery [9]. In addition, some research revealed that levan could have a specific interaction with a certain type of cell, which suggests that this polymer may have the capacity to deliver substantial quantities of a drug to the specific target [14]. In order to produce QA-levan, the effect of GTMAC concentration on QA substitution was investigated (Table 1). The CHN analysis was carried out so that we could verify that the incorporation of trimethylammonium groups into the levan backbone had been successful. In addition, the result demonstrated that QA substitution was increased with the increase in GTMAC concentration. However, at 20% and 40% GTMAC, N/C ratio of the obtained QA-levan were not different. According to this result, 20% GTMAC should be an appropriate amount for preparation of DNA binder.

**Figure 1:**
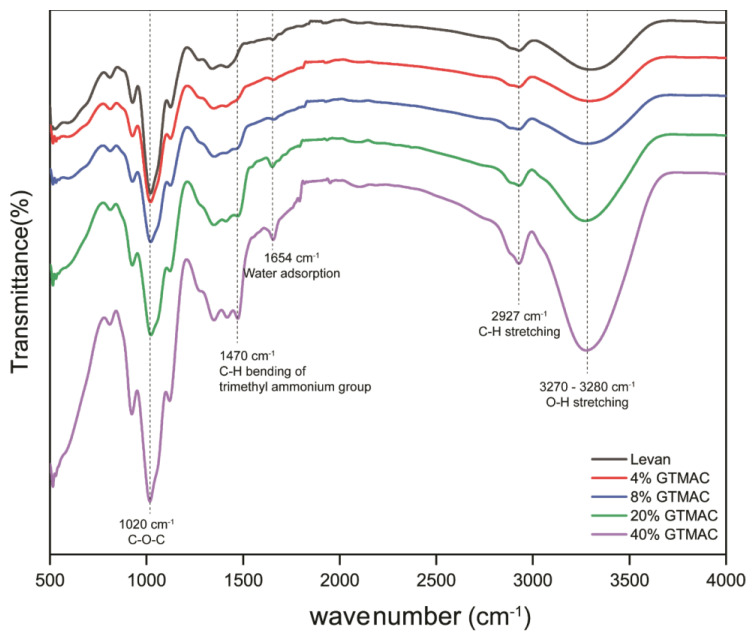
The FT−IR spectra of levan and QA−levan obtained from different GTMAC concentration.

**Figure 2:**
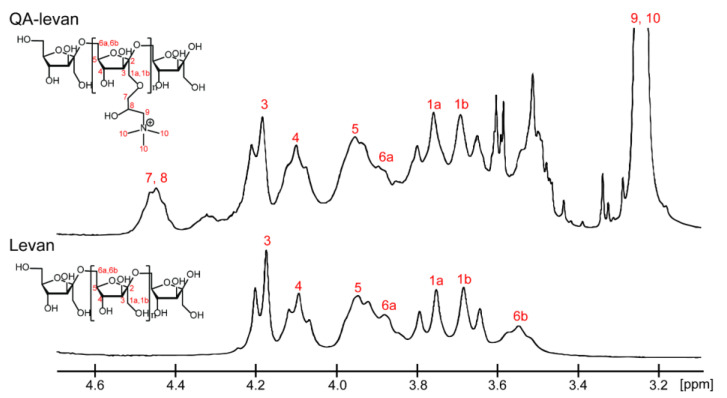
^1^H NMR spectra of levan and QA-levan in D_2_O. Numbers 2−6 represented the H group on the fructosyl unit of levan. Numbers 7−10 were assigned to represent the proton in GTMAC. The data were analyzed using Bruker TopSpin 4.0 software.

The obtained QA-levan is easily soluble in water, and no colloidal particles were seen after dissolution. Trimethylammonium groups, when grafted onto nano-levan, have the potential to increase levan’s water solubility though simultaneously reducing the number of intramolecular contacts that have an effect on self-assembly process. The structure of the obtained QA-levan was analyzed by Fourier-transform infrared (FT-IR) and proton nuclear magnetic resonance (^1^H NMR) spectroscopy. FT-IR spectra showed the signal at ~3300 and ~2900 cm^−1^ which corresponded to O–H and C–H stretching of polysaccharides. The peak at 1654 cm^−1^ is due to water adsorption. However, GTMAC-modified levan displays the additional signal at 1470 cm^−1^ (Figure 1) which is attributable to C–H bending of methyl groups in glycidyl trimethylammonium chloride, confirming the substitution with QA groups on the levan [24]. ^1^H NMR spectrum of QA-levan, in comparison to levan, showed the strong signal around 3.25 ppm. This result reveals the existence of methyl groups in the quaternary ammonium side chains (Figure 2) [28].

### 2.2. Formation of QA−Levan/DNA Polyplexes

**Figure 3:**
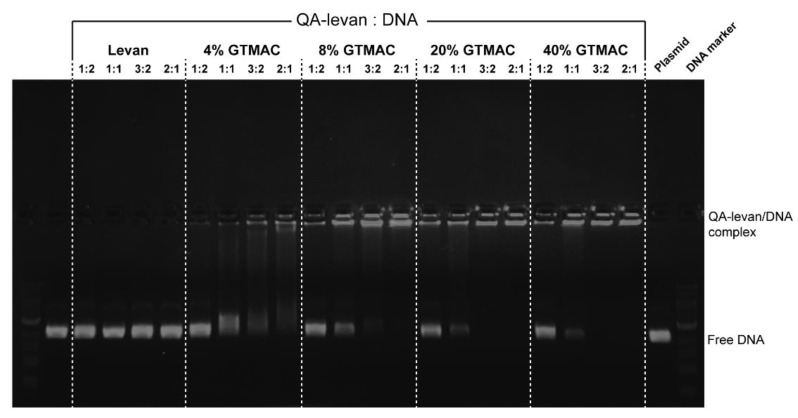
Gel Electrophoresis image of the levan and QA−levan (0.1–0.4 μg) synthesized by different concentration of GTMAC bound to pBlueScript II SK (-) plasmid (0.2 μg).

The cationized QA−levan would form the stable polyplexes with anionized macromolecules, such as DNA, RNA and protein. Nevertheless, the degree of substitution of QA groups in levan molecule ought to be the key factor in that determine the capacity of a cationized levan to bind with nucleic acids. Hence, the formation of QA−levan/DNA polyplexes was examined using differently prepared QA−levan combined with different amount of pBlueScript II SK (-) plasmid. Agarose gel electrophoresis (AGE) was used to monitor the free and bound DNA in solution. According to the results of the AGE, the reaction that was carried out using a higher concentration of GTMAC was able to produce cationized levan with a greater DNA binding affinity. In comparison with native levan, polyplex formation between polysaccharide and DNA was not observed. At 4%GTMAC, DNA migration was still detected in AGE although the ratio of QA−levan:DNA increase up to 2:1, indicating that the QA−levan that was obtained has a relatively weak binding affinity for DNA (Figure 3). Meanwhile, no gel migration was observed for the ratio of 3:2 for 8−40% GTMAC QA−levan. In addition, at a ratio of 2:1 QA−levan/DNA, the QA−levan from 20% and 40% GTMAC exhibits a lower intensity of free DNA bands than those from 8% GTMAC. According to this data, the DNA binding affinity of the QA−levan that was synthesized from 20−40% GTMAC was the best. In addition, it is possible to conclude that the DNA plasmid was not degraded or fragmented because the AGE analysis showed no smeared bands after loading, proving the usefulness of QA−levan as a plasmid protector. In comparison to previous observed, QA−arabinoxylan was totally able to bind to DNA at a ratio of 15:1 (QA−AX:DNA) [25], while QA−dextran was able to entirely bind to DNA when the weight ratio of polymer to DNA was 2:1 [29], suggesting that the QA−levan that was synthesized for this study has a high affinity to form stable QA−levan/DNA nanoplex.

**Figure 4:**
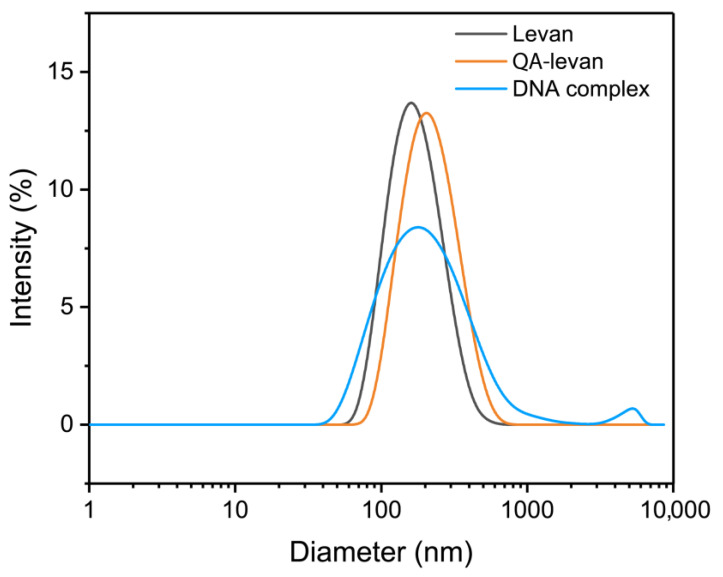
Particle size distribution of native levan, QA−levan and QA−levan/DNA polyplex determined by dynamic light scattering (DLS).

After that, the dynamic diameter of levan, QA-levan and QA−levan/DNA polyplexes were analyzed by dynamic light scattering (DLS) (Figure 4, Table 2). The results showed that levan formed monodisperse nanoparticles with average hydrodynamic diameters of 157.9 ± 0.2 nm, which corresponded to previous observe [9]. After treated with GTMAC, the size of obtained QA−levan is increased to 193.8 ± 1.5 nm. These monodisperse DLS signals that observed in both levan and QA−levan conclusively demonstrate that aggregation of nanoparticles did not occur in aqueous environments. The increase in average diameter of the QA−levan presumably because it is more swollen compared to levan. QA group on the levan chain could strongly interact with water molecules, that may interfere with the self-assembly process by altering the nature of the interactions involved. Zeta potential values clearly demonstrated that the net charge of QA−levan is positive (Table 2), and thus it should strongly bind to the negative macromolecules. Nevertheless, it was found that the formation of QA−levan/DNA polyplexes reduce the size of nanoparticles to 170.7 ± 1.7 nm (Figure 4, Table 2). In addition, the signal of large aggregates was observed. This finding indicates that ionic interaction between DNA and cationized levan may shield the electrostatic repulsion between positive charge group of QA group, and also increase the packing of the polymer.

Previously, the number of positive−charged polysaccharides have been generated using chemical reaction. The size of cationized nanoparticle is varied that is dependent on the type of polysaccharide and modification method used. For example, the particle diameter of positively charged cycloamylose produced by the introduction of (3-Chloro-2 hydroxypropyl) trimethylammonium chloride (CAQ) was 193.2 ± 161.2 nm and could be increased to 379.0 ± 156.7 nm after forming a polyplex with siRNA [30], whereas diethylenetriamine-functionalized Inulin (Inu-DETA) combinded with siRNA exhibited larger size up to 700 nm [31]. The diameter of QA-dextran/DNA polyplex can be range from around 160 to 270 nm, depending on polymer:DNA weight ratio combined [29]. QA-pullulan combined with miRNA has a diameter around 133 nm [24]. However, the size of nanoparticle should be dependent on the size of parent polysaccharide used. In comparison to other studies, the average size of QA−levan in this study was smaller than that seen in some previous research.

### 2.3. Drug Delivery Application of QA−Levan

**Figure 5:**
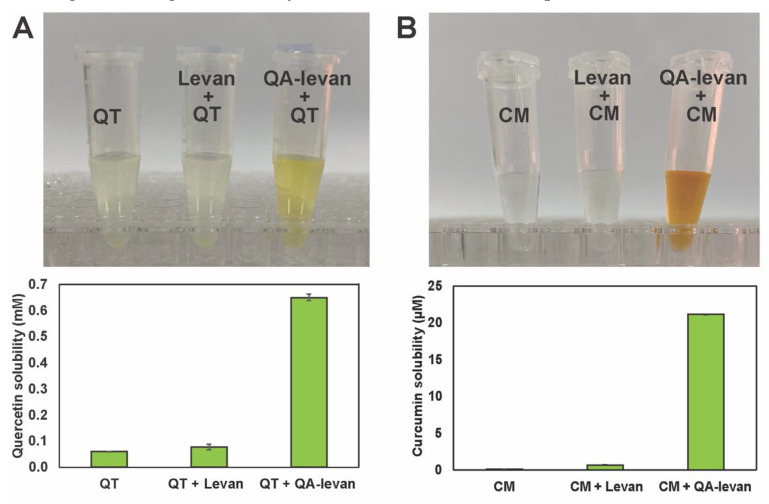
Water solubility of (**A**) quercetin (QT) and (**B**) curcumin (CM) complexed with levan and QA-levan.

The use of nanoparticles that are produced from bacterial exopolysaccharides (EPS) shows potential as a drug carrier as they have the ability to make the medicine more soluble and stable [10]. However, these polysaccharides have a low drug-loaded capacity, hence their use in drug delivery is constrained. Chemical modification might alter the surface properties of these nanoparticles, which could have an impact on how ligands and biopolymers interact. In this study, the ability of QA-levan to form the complex with drugs was demonstrated using quercetin (QT) and curcumin (CM). Quercetin is a flavonoid that displays excellent antioxidant, anti-inflammatory [32], anti-mutagenic and/or anti-carcinogenic activity [33]. It also has the ability to inhibit a number of enzymes that play important roles in health. Curcumin is bioactive compound mostly found in turmeric, which possesses potent anti-oxidant, anti-cancer as well as anti-inflammatory properties [34]. Due to the fact that both quercetin and curcumin have a low bioavailability and is water-insoluble, the use of these drugs is restricted. As shown in Figure 5A,B, QA-levan significantly improved the solubility of QT and CM by 11-folds and 205-folds compared to dissolving in water, respectively. This result suggested another application of QA-levan as a drug carrier. It’s possible that the drug would bind to the alkyl chain of GTMAC via hydrophobic interaction or bind to fructosyl units via hydrogen bond, despite the fact that the net positive charge was provided by the quaternary ammonium groups of GTMAC. Formerly, a wide variety of drug carrier systems have been developed in order to enhance the solubility and bioavailability of these drugs. For instance, the solubility of curcumin increased up to 110-fold after binding with nanocellulose/PVA [35]. Chitosan-grafted pNIPAM copolymer nanogel has ability to improve the solubility of curcumin up to 18-fold compared to the free drug in PBS [36]. The solubility of quercetin is improved by 4.3-fold after combining with nano-inulin [10]. The solubility of quercetin is increased by 2.5-fold compared to the free drug in water when glucan derived from *Leuconostoc holzapfelii* KM01 is used as a carrier [37]. However, in comparison to QA-levan, the improvement in drug solubility that can achieve through the use of these approaches is still quite low. Also, levan had the ability to selectively attach to cells that over-express the GLUT5, such as breast cancer cells. Therefore, QA-levan has the potential to be utilized in applications relating to the targeted delivery of nucleic acids as well as pharmaceuticals.

### 2.4. Cytotoxicity to Human Cancer Cell Line

**Figure 6:**
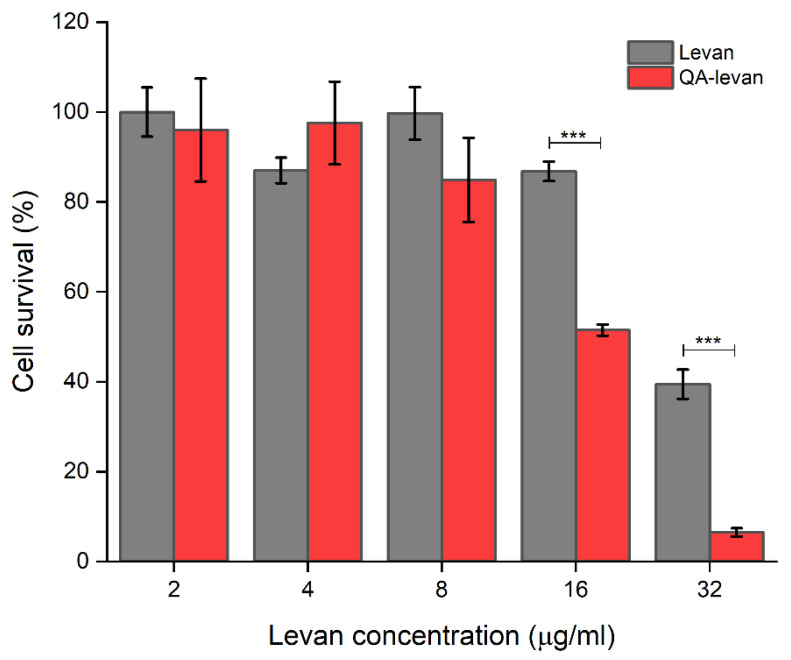
Cytotoxicity of native levan and QA-levan toward HEK293 cells measured by MTT assay. Each data point is presented as mean ± SEM (*** *p* < 0.001 vs. control).

Many essential cellular functions can be inhibited by non-specific interactions between highly charged cationic polymers and anionic biomolecules in the cellular system. To determine if the developed QA-levan may be employed in biomedical applications, specifically as vectors for gene therapy, the cytocompatibility towards cell lines was examined in this study using MTT assay (Figure 6). The result revealed that both levan and OA-levan were not toxic at the concentration range of 2–8 µg/mL. However, at 16 µg/mL, the QA-levan (IC_50_ = 18.3 µg/mL) significantly suppressed the growth of HEK293 cells compared to native levan (IC_50_ = 28.5 µg/mL). Due to the fact that levan has its own anticancer activity [9], cationization may boost the levan’s anticancer effect. The positively charged groups on cationized compounds can interact with negatively charged molecules on the cell surface, increasing binding and uptake into the cell. In contrast, anionized compounds can repel the negatively charged molecules on the cell surface, making it difficult for the anionized compound to bind and enter the cell. As a result, cationized compounds often have a higher susceptibility for cellular uptake than anionized compounds [38,39], resulting in a high level of toxicity towards an immortalized human embryonic kidney cell, HEK293. However, in comparison to the well-known transfection agent such as DEAE-dextran, QA-Levan showed a comparable cytotoxicity (IC_50_ ~ 8–10 µg/mL) [40]. Although hazardous at high concentrations, QA-levan at non-toxic concentration should bind adequate DNA for transfection. Drug delivery application of QA-levan requires careful consideration of multiple factors, including the dose and duration of exposure in order to find the appropriate balance between a polymer’s toxicity and efficacy in drug delivery. Moreover, the toxicity of the polymers towards different cell lines must be studied in greater detail before using them for cellular uptake applications. As a result, the QA-levan polymer has the potential to be a versatile material that serves as a carrier for both drugs and genes. This study also demonstrated a further application of bacterial exopolysaccharide in biomedicine. 

## 3. Conclusions

In this study, the cationized nanolevan was prepared by chemical modification using glycidyl trimethylammonium chloride (GTMAC). The QA-levan obtained demonstrates a potentially useful capacity to bind to hydrophobic substances, quercetin and curcumin, as well as plasmid DNA. It was investigated how the concentration of GTMAC affected the ability of QA-levan to form a nanoplex with DNA. Agarose gel electrophoresis showed that the 40% GTMAC is an optimum condition for preparing DNA binder. On top of that, the cationize OA-levan could increase the solubility of quercetin and curcumin in water by 11-folds and 205-folds compared to dissolving in water, respectively. In comparison to some other system, QA-levan showed significant improvement to increase quercetin and curcumin solubility. The results of this study provide more evidence that GTMAC-modified levan may be useful for the transportation of both pharmaceuticals and nucleic acids.

## 4. Materials and Methods

### 4.1. Chemicals

Glycidyl trimethylammonium chloride (GTMAC) was purchased from Tokyo Chemical Industry (TCI). Sucrose was purchased from UNIVAR. Potassium dehydrogenase phosphate (KH_2_PO_4_) and di-potassium hydrogen phosphate (K_2_HPO_4_) was purchased from KEMAUS TOYOPEARL™ AF-Chelate-650M used for Ni-affinity chromatography was obtained from Tosoh bioscience. Quercetin and curcumin were obtained from Sigma-Aldrich. Dimethyl sulfoxide (DMSO) was purchased from Merck. Dulbecco’s Modified Eagle Medium (DMEM) for cell culture was obtained from Thermo Fisher Scientific (Waltham, MA, USA). MTT (3-(4,5-Dimethylthiazol-2-yl)-2,5-Diphenyltetrazolium Bromide) was purchased from Sigma-Aldrich.

### 4.2. Preparation of Levan

Levan was synthesized by recombinant levansucrase from *E. tasmaniensis*. The recombinant levansucrase was expressed *in E. coli* BL21 (DE3) with Isopropyl β-D-1-thiogalactopyranoside (IPTG) induction, and then purified by Ni-affinity chromatography according to the previous report [9]. After that, 5 U/mL of purified levansucrase was added to substrate solution containing 20% (*w/v*) sucrose and 50 mM potassium phosphate buffer (pH 6.5). The reaction mixture was incubated at 30 °C for 24 h. The obtained levan polymer was separated from the reaction mixture by acetone precipitation. Two volumes of acetone were added to the reaction mixture, mixed by shaking and kept at −20 °C for overnight. Levan was separated by centrifugation at 10,000× *g* for 20 min. The obtained precipitant was resuspended in DI water and dialyzed against DI water. The resulting levan suspension was lyophilized and kept at room temperature.

### 4.3. Preparation of Quaternary Ammonium Levan (QA-Levan)

**Figure 7:**
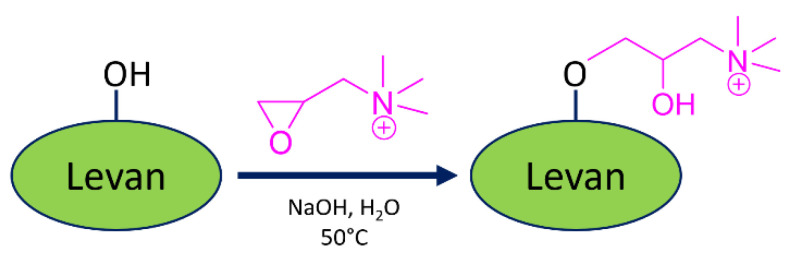
Synthetic scheme for cationized levan using Glycidyl trimethylammonium chloride (GTMAC).

The cationization of levan was accomplished by reacting levan with glycidyl trimethylammonium chloride (GTMAC) under alkaline condition as illustrated in Figure 7. Two hundred milligrams of levan was dissolved in 5 mL of 12.5% (*w/v*) of NaOH solution. The reaction was incubated in shaking incubator at 25 °C for 4 h. After that, GTMAC was added to levan solution to final concentration of 4–40%. The reaction was then performed at 50 °C overnight. Then, the modified levan (QA-levan) was separated from the reaction mixture by ethanol precipitation. Two volumes of cold ethanol (~10 mL) were added to the reaction mixture, stayed at −20 °C overnight, and centrifuged at 10,000× *g* for 30 min. The obtained polymer in precipitate was dissolved in 5 mL DI water and dialyzed against DI water until the pH value became neutral. Finally, the obtained QA-levan was lyophilized and kept at room temperature until further analysis.

### 4.4. Characterization

#### 4.4.1. Fourier Transform Infrared (FT-IR) Analysis

The FTIR spectra of native and modified nanolevan were recorded using FTIR spectrometer (Perkin Elmer, Spectrum Two). FTIR spectrum were recorded in the region of 500–4000 cm^−1^ with a resolution of 16 cm^−1^.

#### 4.4.2. CHN Analysis

The element compositions of native and modified levan were characterized by CHNS elemental analyzer (Thermo Fisher Scientific, FlashSmart) according to methodology described in ISO 16948:2015.

#### 4.4.3. ^1^H-NMR

Approximately 30 mg of native and modified levan was dissolved in D_2_O. The ^1^H NMR spectra of native levan and QA-levan were acquired using a Bruker Avance-300 spectrometer at 25 °C. The data were analyzed using Bruker TopSpin 4.0 software.

#### 4.4.4. Zeta Sizer

The zeta potential and nanoparticle size of the levan and its derivative were recorded by Dynamic light scattering (DLS) particle size analyzer (Malvern Panalytical, Zetasizer Nano) using disposable folded capillary cell. The samples were resuspended in DI water at 25 °C, and filtered thought 0.45 micron filter before analysis.

### 4.5. Formation of QA-Levan/DNA Polyplexes

1 mg of QA-levan was initially dissolved in 1 mL ultrapure water. Subsequently, pBlueScript II SK (-) plasmid was combined with cationized levan solution to the mass ratio of 0.1 μg levan and 0.2 μg plasmid (1:2), 0.2 μg levan + 0.2 μg plasmid (1:1), 0.3 μg levan + 0.2 μg plasmid (3:2) and 0.4 μg levan + 0.2 μg plasmid (2:1), and stayed at room temperature for 5 min. The formation of QA-levan/DNA polyplexes was analyzed by 1% (*w/v*) agarose gel electrophoresis in TAE buffer stained with EcoDye™ DNA staining solution (BIOFACT, Daejeon, Republic of Korea). The bands of free DNA and QA-levan/DNA polyplexes in gel electrophoresis was monitored by Amersham ImageQuant™ 800 (Marlborough, MA, USA).

### 4.6. Encapsulation of Small Molecules

The binding of small molecules, including quercetin (QT) and curcumin (CT), on the levan and QA-levan were investigated. An excess amount of QT or CM was added to the 2% (*w/v*) levan or QA-levan solutions and consequently incubated in shaking incubator at 200 rpm, 30 °C for 8 h. The insoluble drugs were removed from the solutions by means of centrifugation at a speed of 12,000× *g* for 10 min. The supernatant was collected, and concentration of entrapped QT and CM was determined by spectrophotometer at 380 nm and 427 nm, respectively, using QT and CM as standards.

### 4.7. Cytotoxicity

Cytotoxicity of levan and QA-levan was evaluated in HEK293 cells using MTT assay [41]. HEK293 cells (5000 cells per well) were seeded in 96-well plates and cultured in complete Dulbecco’s Modified Eagle Medium (DMEM) at 37 °C for 16 h under 5% CO_2_. The cells were subsequently treated with levan or QA-levan at concentration of 2, 4, 8, 16 and 32 µg/mL for 72 h. After that, 10 µL of MTT reagent was added to each well and incubated at 37 °C for 3 h. 100 µL of DMSO was added to the reaction for dissolving the purple formazan crystals. Absorbance was measured at 570 nm using a microplate reader (Biotek Model Synergy H1). Cell viability (%) was computed by comparison with untreated cells. 

## Figures and Tables

**Table 1:** CHN analysis of QA-levan obtained from different condition.

Sample	%N	%C	%H	N/C
Levan	ND	42.29	6.92	ND
4%GTMAC modified levan	0.70	39.59	6.46	0.02
8%GTMAC modified levan	1.24	40.32	6.65	0.03
20%GTMAC modified levan	2.86	41.03	7.34	0.07
40%GTMAC modified levan	2.45	36.72	6.92	0.07

**Table 2:** Diameter and surface charge density of levan, QA-levan and polyplex.

Sample	Diameter (nm)	ξ-Potential Mean ± SEM (mV)
Levan	157.9 ± 0.2	−7.05 ± 0.19
QA-levan	193.8 ± 1.5	53.7 ± 0.53
QA-levan/DNA complex	170.7 ± 1.7	47.9 ± 0.70

## Data Availability

Data is contained within the article.
